# Hydroxychloroquine/chloroquine and the risk of acute kidney injury in COVID-19 patients: a systematic review and meta-analysis

**DOI:** 10.1080/0886022X.2022.2046609

**Published:** 2022-03-07

**Authors:** Zheng-Ming Liao, Zhong-Min Zhang, Qi Liu

**Affiliations:** a Department of Urology, Hubei Provincial Hospital of Traditional Chinese Medicine, Wuhan, China; b Affiliated Hospital of Hubei University of Chinese Medicine, Wuhan, China; c Hubei Province Academy of Traditional Chinese Medicine, Wuhan, China

**Keywords:** Hydroxychloroquine, COVID-19, acute kidney injury, meta-analysis

## Abstract

**Objectives:**

Hydroxychloroquine/chloroquine has been widely used as part of the standard treatment for patients with coronavirus disease 2019 (COVID-19). We conducted a systematic review and meta-analysis to determine whether hydroxychloroquine/chloroquine increases the risk of acute kidney injury (AKI) in COVID-19 patients.

**Methods:**

PubMed and Embase were searched for related publications from inception to Dec 31, 2021, including randomized controlled trials (RCTs) and non-randomized studies of interventions (NRSIs) comparing the risk of AKI and/or increased creatinine in COVID-19 patients receiving hydroxychloroquine/chloroquine and other controls (active treatment and placebo). We conducted separate meta-analyses for RCTs and NRSIs based on fixed-effect model, with odds ratios (ORs) being considered as effect sizes.

**Results:**

We included 21 studies in the analysis, with 12 were RCTs. Based on the RCTs, compared to placebo, the OR was 1.19 (95% confidence interval [CI]: 0.86, 1.64; *p* = .30, *n* = 4, moderate quality) for AKI and 1.00 (95%CI: 0.64, 1.56; *p* = .99, *n* = 5, moderate quality) for increased creatinine for patients received hydroxychloroquine/chloroquine treatment; compared to active treatment, the odds was 1.28 (95%CI: 0.65, 2.53; *p* = .47, *n* = 2, low quality) for AKI and 0.64 (95%CI: 0.13, 3.20; *p* = .59, *n* = 1, low quality) for increased creatine. Evidence from NRSIs showed slightly increased odds of AKI, with low quality.

**Conclusion:**

Based on current available studies which were graded as low to moderate quality, there is insufficient evidence to conclude that hydroxychloroquine/chloroquine use is associated with increased risk of AKI or raised creatinine. **Abbreviations:** AKI: acute kidney injury; COVID-19: Coronavirus Disease 2019; RCT: randomized controlled trials; NRSI: non-randomized studies of interventions; OR: odds ratios; ROBIS-I: Risk Of Bias In Non-randomized Studies – of Interventions

## Introduction

The coronavirus disease 2019 (COVID-19) pneumonia is caused by a new type of coronavirus, posing a huge threat to human health. According to the World Health Organization Coronavirus Dashboard, there have been 230,418,451 confirmed cases and 4,724,876 deaths globally, as of September 24, 2021 [1]. The common symptoms of COVID-19 pneumonia include fever, cough, headache, and other influenza-like symptoms [2]. While increasing evidence suggests that many COVID-19 patients have complications in the urinary system, acute kidney injury (AKI) is one of the most severe conditions [3–5]. As estimated by a meta-analysis, about 11% of the COVID-19 patients would develop AKI over 2 to 28 days follow-up and 6.8% needed the renal replacement therapy [6].

AKI is defined as a sudden loss of excretory kidney function that occurs within a few hours or days, presenting as an increase in serum creatinine levels with or without reduced urine output [7]. Many factors can lead to AKI, such as decreased renal perfusion/arterial pressure by any other conditions (e.g., heart failure), damage to tubular cells by nephrotoxic agents, or acute inflammation of blood vessels and glomeruli by systemic illness [8,9]. For COVID-19 patients, it is still unclear if AKI is largely due to hemodynamic changes, nephrotoxic agents or if the virus also leads to direct cytotoxicity [10].

Hydroxychloroquine, a derivative of chloroquine that has both antimalarial and anti-inflammatory activities, has been considered as part of the standard care for COVID-19 patients in some countries, especially during the first pandemic phase [11,12]. Although existing well-conducted meta-analyses have denied the potential benefits of hydroxychloroquine/chloroquine treatment on the prognosis of COVID-19 patients [12–15], millions of patients have already received such treatment. Hydroxychloroquine was found could potentially induce or intensify AKI by increasing lysosomal pH and inhibiting autophagy, because it has been demonstrated that Hydroxychloroquine inhibits autophagic flux by impairing autophagosome-lysosome fusion [16]. However, epidemiological evidence suggested controversy findings that make it unclear whether the development of AKI was associated with the use of hydroxychloroquine/chloroquine.

In this study, we conducted a systematic review and meta-analysis of current studies to explore the relationship between hydroxychloroquine/chloroquine and the risk of AKI.

## Methods

The current systematic review was registered in PROSPERO (CRD42021265663) and was conducted and reported according to the Preferred Reporting Items for Systematic reviews and Meta-Analyses statement 2020 [17].

### Inclusion criteria

We included original studies that investigated the relationship between hydroxychloroquine/chloroquine treatment and the risk of AKI in COVID-19 patients (both adults and children). The primary intervention was hydroxychloroquine or chloroquine, while the control was any other treatment meant for active treatments, supportive care, or placebo. The primary outcome was AKI reported by the original studies, and the secondary outcome was increased creatinine. For the study design, we considered randomized controlled trials (RCTs), cohort studies, and case-control studies. For simplicity, we referred to the latter two types of studies as non-randomized studies of intervention (NRSIs). For studies with few patients who may have had renal failure before the infection, we only considered new cases of AKI after treatment.

### Literature search

We searched PubMed and Embase for studies on the treatment of COVID-19 patients with hydroxychloroquine/chloroquine published from inception to July 10, 2021 (LZ). An updated search on Dec 31, 2021 was conducted during the process of revision. We did not search for unpublished sources because the current largest preprint database for health science (medRxiv) with the topic of COVID-19 has already been indexed in PubMed. We did not restrict the outcome of the search strategy to avoid potential omissions for eligible studies. The following medical subject headings and/or keywords were used for hydroxychloroquine/chloroquine: ‘Hydroxychloroquine’, ‘Oxychloroquine’, ‘Plaquenil’, ‘Chloroquine’, ‘Chlorochin’, ‘Chingamin’, ‘Khingamin’, ‘Nivaquine’, ‘Aralen’, and ‘Arechine’. Moreover, the following medical subject headings and/or keywords were used for COVID-19: ‘COVID-19’, ‘2019-Cov’, ‘2019-nCov’, ‘nCov’, ‘severe acute respiratory syndrome coronavirus 2’, ‘SARS-CoV-2’, ‘sars coronavirus 2’, ‘SARS-2’, ‘SARS2’, ‘2019-novel-corona’, ‘Novel-corona’, ‘novel coronavirus’, ‘New-corona’, ‘Coronavirus’, ‘coronavirus 2’, ‘corona virus’, ‘Betacoronavirus’, and ‘Cov2’. The full search strategy is presented in the Supplementary Appendix.

### Literature screen

Two review authors (LZ, ZZ) independently conducted the literature review. We first screened the titles and abstracts of the publications obtained by the search, followed by a review of the full text of the remaining records. Any disagreements were resolved through discussion. For each potential study, we also checked ClinicalTrial.gov for the outcome information.

### Data extraction

Two review authors (ZZ, LQ) conducted data extraction using a pre-specified Excel sheet template. The following information was extracted: name of the first author, country where the study was conducted, patient characteristics (including age, comorbidity, diagnostic methods), intervention agent and dosage, control agent and dosage, outcomes, sample size of the study, number of outcomes in each group, follow-up of the study for the outcomes, and type of studies. Any disagreements in the extracted information were resolved through discussion.

### Risk of bias

As planned, we used the Cochrane ROB 2.0 tool to assess the RCT’s risk of bias [18]. This tool considers five domains: bias arising from the randomization process, bias due to deviations from intended interventions, bias due to missing outcome data, bias in the measurement of the outcome, and bias in the selection of the reported result. For the cohort and case-control studies, we primarily planned to use the Newcastle-Ottawa Scale checklist [19], but we found that it could not be presented through a risk of bias plot. Therefore, we used the Risk Of Bias In Non-randomized Studies - of Interventions (ROBIS-I), as suggested by the Cochrane handbook, to assess the risk of bias [20]. To reduce subjective judgment, all review authors assessed the risk of bias.

### Statistical analysis

The meta-analyses were conducted using RevMan 5.3 (Cochrane; London, United Kingdom) and Stata 12.0 (StataCorp LLC; Texas, United States). Given that both the primary and secondary outcomes were binary outcomes, we used the odds ratio (OR) and its 95% confidence interval (CI) to measure the potentially harmful effects of hydroxychloroquine/chloroquine. Furthermore, we used the Mantel-Haenszel method to pool the study-specific effects [21], and studies with different designs (RCTs vs. NRSIs) were pooled separately. Statistical significance was set at *p* < .05. Considering that the events of AKI and increased creatinine could be rare in some of the studies, we neglected the potential heterogeneity among the included studies and used the fixed-effect model to increase the statistical power. This decision was based on the recommendations of the Cochrane Handbook [21]. Considering the potential zero events in some studies, we used risk difference based on the Mantel-Haenszel method as a sensitivity analysis for those with double-zero studies [22].

Heterogeneity was measured using I^2^. As suggested in the Cochrane handbook [21], we treated I^2^ < 30% as no or slight heterogeneity; otherwise, there was moderate or substantial heterogeneity (I^2^ ≥ 30%). For meta-analyses with moderate or substantial heterogeneity, the evidence was downgraded and a sensitivity analysis using a random-effect model based on the inverse-variance method was conducted.

Subgroup analysis was performed by type of control to determine if there was a difference in the ORs for active and placebo controls. We primarily planned to use age and dosage of treatment for the subgroup analysis, but half of the included studies did not provide detailed age information (i.e., adults or children), and only a few studies used different doses, making the planned subgroup analyses unimplementable. A further sensitivity analysis was conducted by omitting one study at a time to determine if the results were stable. Egger’s method was used to detect potential publication bias. Finally, the Grading of Recommendations Assessment, Development and Evaluation tool was used to rate the global evidence of each meta-analysis to form the conclusions [23].

## Results

### Baseline characteristics

The initial literature search in the two databases resulted in 4,830 records, and we identified 798 duplicates by author’s name, title, and publication year. Therefore, 4,032 publications were screened by title and abstract. We further excluded 3,832 records that did not meet the criteria, and the remaining 200 publications were screened through a review of the full text. After excluding 134 with different outcomes, 33 with different interventions, ten with different populations, and three with different study types, we finally included 20 studies [24–43] for the current systematic review, with a kappa value of 0.85 between the two reviewers (Figure S1, Supplementary Appendix). The updated search in 31, Dec 2021 (10 July, 2021 to 31, Dec 2021) resulted in 648 records, 72 were identified as duplicates, and 1 RCT [44] was identified as new studies that included in current meta-analysis.

**Figure 1.**
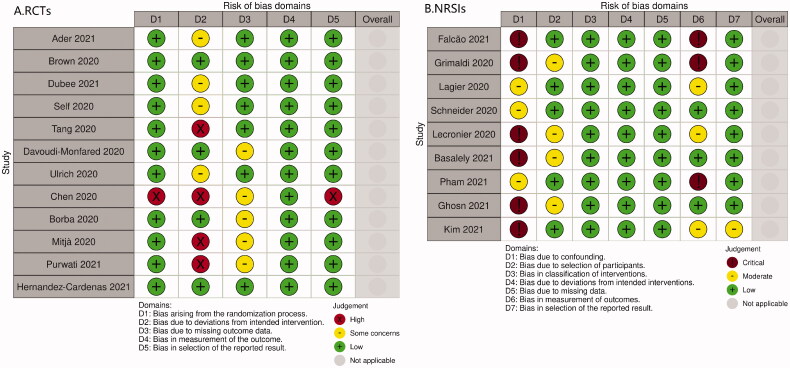
The risk of bias of included studies.

Table 1 presents the main characteristics of the included studies. Among the 21 included studies, 14 considered AKI outcomes, and seven analyzed increased creatinine levels. Of the 14 studies on AKI, six were RCTs, six were cohort studies, and two were nested case-control studies. For the seven studies on increased creatinine levels, six were RCTs, and one was a cohort study. For the 21 studies, 13 clearly specified the age range, with 12 focusing on adults and one focusing on children. The daily total dosage of hydroxychloroquine/chloroquine ranged from 200 mg to 1200 mg (available from 18 studies), with 13 studies providing 400 mg, two studies providing 200 mg, one study providing 600 mg, one study providing 800 mg, and one study providing 1200 mg. The follow-up for the safety outcomes ranged from 3 to 42 days, while three studies did not provide such information. Eight studies were conducted in Europe, seven in Asia, and six in America.

**Table 1.** Baseline characteristics of included studies.

Author	Country	Population	Age (years, IQR)	Intervention (maintain dose)	Comparison (maintain dose)	Outcomes (total)	Sample size (events)	Follow-up for safety	Type of study
Ader 2021 [24]	France	Adults (≥18 years) with a PCR-proven SARS-COV-2 infection, 71.7% male; patients with Stage 4 severe chronic kidney disease or requiring dialysis (i.e., eGFR <30 mL/min) were excluded	63 (54 to 71)	Hydroxychloroquine (400 mg once daily) plus Standard care	Standard care	AKI: 50 out 579 patients	143 (14) vs. 148 (9)	29 days	Randomized controlled trial
					Lopinavir (400 mg twice daily)/ritonavir (100 mg twice daily) plus Standard care		143 (14) vs. 144 (16)		
Brown 2020 [25]	America	Hospitalized patients with symptomatic laboratory-confirmed COVID-19 within 10 days of a positive test; patients with renal failure were excluded	55 (42 to 65)	Hydroxychloroquine (200 mg twice daily)	Azithromycin (250 mg daily)	AKI: 6 out of 83 patients	41 (6) vs. 42 (0)	5 days	Randomized controlled trial
Dubee 2021 [26]	France	Adult (aged ≥18 years) with a diagnosis of COVID-19 confirmed by positive SARS-CoV-2 RT-PCR test on a nasopharyngeal swab;	77 (58 to 86)	Hydroxychloroquine (200 mg twice daily)	Placebo	AKI: 131 out of 244 patients	124 (70) vs. 120 (61)	28 days	Randomized controlled trial
Self 2020 [27]	Iran	Adults (aged ≥18 years) who were hospitalized for less than 48 hours with laboratory-confirmed SARS-CoV-2 infection and symptoms of respiratory illness for less than 10 days were enrolled	57.5 (43 to 69)	Hydroxychloroquine sulfate (200 mg twice daily)	Placebo	AKI: 74 out of 579 patients	242 (37) vs. 237 (37)	28 days	Randomized controlled trial
Tang 2020 [28]	China	Adult (aged ≥ 18 years) with a diagnosis of COVID-19 confirmed by RT-PCR; patients with severe conditions including kidney disease and renal impairment were excluded	46.1 (SD: 14.7)	Hydroxychloroquine (800 mg daily) plus Standard of care	Standard of care	KI: 1 out of 150 patients	70 (1) vs. 80 (0)	28 days	Randomized controlled trial
Davoudi-Monfared 2020 [29]	Iran	Adult (aged ≥18 years) patients diagnosed with COVID-19; 3 patients (2 vs. 1) with renal disease secondary to COVID-19 was included.	61 (50 to 70)	Hydroxychloroquine (200 mg twice daily) plus lopinavir-ritonavir (400 and 100 mg, respectively, twice daily) or atazanavir-ritonavir	IFN -1a (44 ug/ml)	AKI: 23 out of 81 patients	39 (11) vs. 42 (12)	30 days	Randomized controlled trial
Falcão 2021 [30]	Portugal	Adult (aged ≥18 years) with a diagnosis of COVID-19 confirmed by positive SARS-CoV-2 RT-PCR test; 19 CKD patients in Hydroxychloroquine group and 1 in Remdesivir group were included.	65.5/62 (18∼)	Hydroxychloroquine (200 mg twice daily)	Remdesivir (100 mg once daily)	AKI: 9 out of 149 patients	101 (8) vs. 48 (1)	18 to 22 days	Cohort study
Grimaldi 2020 [31]	Belgian and French	Adult (aged ≥18 years) with a diagnosis of COVID-19 confirmed by positive PCR test with moderate-to-severe acute respiratory distress syndrome	63 (18∼)	Hydroxychloroquine (no dose information)	Lopinavir ritonavir (no dose information)	AKI: 261 out of 414 patients	183 (124) vs. 231 (137)	28 days	Cohort study
Lagier 2020 [32]	Marseille and France	Adult (aged ≥ 18 years) with PCR-documented SARS-CoV-2 RNA from a nasopharyngeal sample	45.3 (18∼)	Hydroxychloroquine (200 mg three times daily) and Azithromycin (250 mg once daily)	Azithromycin (250 mg once daily)	AKI: 4 out of 3737 patients	3337 (4) vs. 137 (0)	3 to 13 days	Cohort study
				Hydroxychloroquine (200 mg three times daily)	Azithromycin (250 mg once daily)		101 (0) vs. 137 (0)		
Schneider 2020 [33]	Germany	Patients with PCR-documented SARS-CoV-2 RNA from a nasopharyngeal or oropharyngeal swab specimen; 5 and 3 with CKD in treatment and control groups; non-ICU sample was used here	67 in treatment and 70.5 in control	Hydroxychloroquine (200 mg twice daily) plus lopinavir/ritonavir	Unclear treatment	AKI: 13 out of 28	14 (11) vs. 14 (2)	NA	Cohort study
Lecronier 2020 [34]	France	Critical III patients with infection by SARS-CoV-2 defined by positive reverse transcriptase polymerase chain reaction (RT-PCR)	57 (53 to 68)	Hydroxychloroquine (200 mg twice daily) plus standard care	Standard care	AKI: 40 out of 80 patients	38 (16) vs. 22 (12)	7 days	Cohort study
				Hydroxychloroquine (200 mg twice daily) plus standard care	Lopinavir/ritonavir (400 mg twice daily) plus standard care		38 (16) vs. 20 (12)		
Basalely 2021 [35]	America	Children (≤18 years) who were admitted for treatment of acute COVID-19; Patients who were kidney transplant recipients, patients with end-stage kidney disease (estimated glomerular filtration rate <15 ml/min per 1.73 m2 or dialysis) were excluded	8.2 (1.5 to 13.8)	Hydroxychloroquine (no dose information)	Without Hydroxychloroquine	AKI: 8 out of 97 patients	8 (3) vs. 88 (14)	NA	Nested case control
Pham 2021 [36]	America	All patients who presented to Weill Cornell Medical Center with confirmed COVID-19 by RT-PCR of nasopharyngeal sample; 2 and 3 with end-stage renal disease at the entry	63 (43 to 73)	Hydroxychloroquine (200 or 400 mg daily)	Without Hydroxychloroquine	AKI: 18 out of 42 patients	14 (9) vs. 28 (9)	28 days	Nested case control
Ghosn 2021 [37]	Abu Dhabi	Adult critical III patients (age ≥ 18 years) admitted to our ICU with confirmed SARS-CoV-2 infection by RT-PCR of nasopharyngeal sample; patients admitted to ICU with reasons other than acute respiratory were excluded.	50 (40 to 59)	Hydroxychloroquine (no dose information)	Without Hydroxychloroquine	AKI: 50 out of 110 patients	50 (18) vs. 60 (27)	NA	Case control
Outcomes were not primary clearly defined as AKI									
Ulrich 2020 [38]	America	Hospitalized patients with a positive SARS-CoV-2 by RT-PCR with at least one symptom	66.2 (SD: 16.2)	Hydroxychloroquine (200 mg daily)	Placebo	Increased creatinine: 7 out of 128 patients	67 (5) vs. 61 (2)	30 days	Randomized controlled trial
Chen 2020 [39]	China	Treatment naïve adult patients (≥ 18 years) confirmed COVID-19; patients with any renal disease were excluded	48.6 (18∼)	Hydroxychloroquine (400 mg once daily) plus standard supportive care	Standard supportive care	Increased creatinine: 1 out of 30 patients	15 (0) vs. 15 (1)	14 days	Randomized controlled trial
Borba 2020 [40]	Brazil	Hospitalized patients with clinical suspicion of COVID-19 (ie, history of fever and any respiratory symptom, eg, cough or rhinorrhea), aged 18 years or older at the time of inclusion; 4 patients with CKD. Patients further confirmed were used here.	51.1 (SD: 13.9)	Chloroquine diphosphate (600 mg twice daily)	Chloroquine diphosphate (450 mg twice daily)	Increased creatinine: 13 out of 27 patients	18 (8) vs. 9 (5)	14 days	Randomized controlled trial
Mitjà 2020 [41]	Spain	Non-hospitalized adult patients with recently confirmed SARS-CoV-2 infection and less than five days of symptoms, with positive PCR test for SARS-CoV-2 by nasopharyngeal swab; those with moderate or severe condition as well as renal failure were excluded	41.6 (SD: 12.5)	Hydroxychloroquine (400 mg once daily)	Without treatment except usual care	Renal and urinary disorders: 1 out of 351 patients	169 (1) vs. 184 (0)	28 days	Randomized controlled trial
Purwati 2021 [42]	Indonesia	Adult (aged ≥ 18 years) with a diagnosis of COVID-19 with mild to moderate symptoms confirmed by PCR swab test; patients with impaired renal functions were excluded.	36.5 (20 to 55)	Hydroxychloroquine (200 mg once daily) plus Azithromycin (500 mg once daily)	Azithromycin (500 mg once daily)	Increased creatinine: 62 out of 751 patients	121 (11) vs. 119 (9)	7 days	Randomized controlled trial
Kim 2021 [43]	Korea	Patients with PCR-documented SARS-CoV-2 RNA from a nasopharyngeal or oropharyngeal swab specimen, treated with lopinavir-ritonavir or hydroxychloroquine for 7 days or more; 1 and 3 patients with CKD in treatment and control groups	64.3 (SD: 15.4)	Hydroxychloroquine (400 mg once daily)	Lopinavir-ritonavir (400 and 100 mg, respectively)	Increased creatinine: 9 out of 28 patients	34 (6) vs. 31 (3)	42 days	Cohort study
Hernandez-Cardenas 2021 [44]	Mexico	Adults aged more than 18 years with COVID-19 confirmed by RT-PCR and lung injury; no patients had kidney disease.	50 (SD: 11) in treatment and 49 (SD: 12) in control	Hydroxychloroquine (200 mg every 12 hours)	Placebo	Increased creatinine: 144 out of 214 patients	106 (69) vs. 108 (75)	30 days	Randomized controlled trial

IQR: interquartile range; SD: standard deviation; CKD: Chronic Kidney Disease; PCR: Polymerase Chain Reaction; RT-PCR: Real time-Polymerase Chain Reaction; ICU: Intensive Care Unit; AKI: acute kidney injury.

### Risk of bias

Figure 1 presents the risk of bias in the 12 RCTs and nine NRSIs. Among the 12 RCTs, the study by Chen (2020) [39] had a high risk of bias in the randomization process, four studies [28,39,41,42] had a high risk of bias due to the deviation from the intended intervention, and one study [39] had a high risk of bias in the selection of the reported result. In addition, four studies [24,26,27,38] had a possible bias of deviation from the intended intervention, and five [29,39–42] presented some concerns regarding the bias of missing outcomes. For the nine NRSIs, six studies [30,31,34,35,37,43] showed a critical bias, three [32,33,36] showed moderate bias due to confounding, and four studies [31,34,35,37] showed moderate bias due to the selection of participants. In addition, three [31,32,36] showed critical bias, and three [32,34,43] showed moderate bias due to the measurement of outcomes, and one [43] showed moderate bias due to the selection of the reported result.

### Hydroxychloroquine vs. placebo

**Figure 2.**
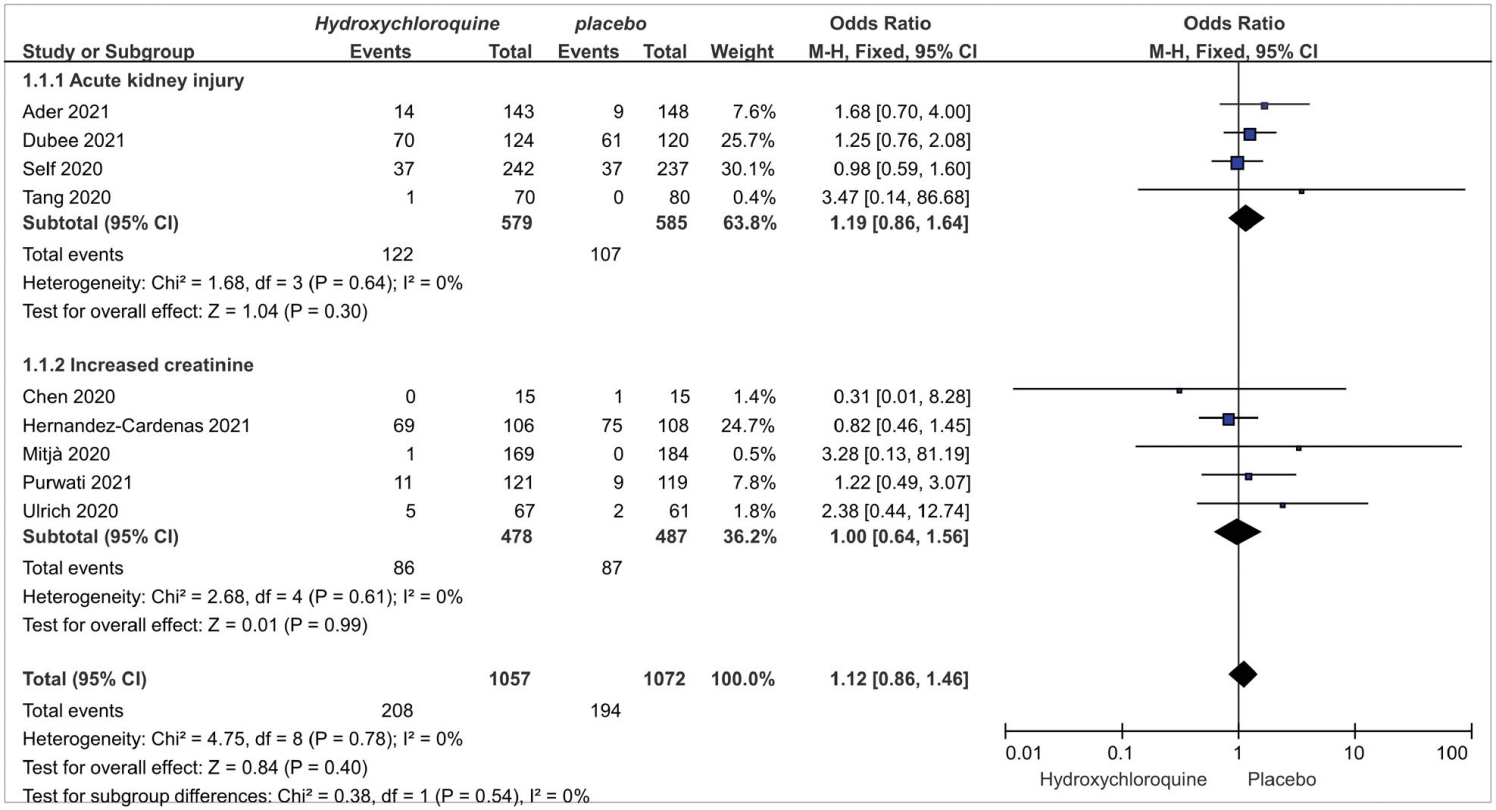
The risk of AKI and increased creatinine for hydroxychloroquine/chloroquine compared to placebo based on the evidence of RCTs.

Figure 2 presents the comparison of hydroxychloroquine/chloroquine vs. placebo on the risk of AKI and increased creatinine in RCTs. The meta-analysis showed that, compared to placebo, the OR of AKI with hydroxychloroquine/chloroquine was 1.19 (95% CI: 0.86, 1.64; *p* = .30, *n* = 4, moderate quality), with no evidence of heterogeneity (I^2^ = 0). Similarly, the OR of increased creatinine was 1.00 (95%CI: 0.64, 1.56; *p* = .99, *n* = 5, moderate quality), with no evidence of heterogeneity (I^2^ = 0). Based on two NRSIs, the OR for AKI was 0.48 (95%CI: 0.16, 0.46, low quality), Figure S2 (Supplementary Appendix).

### Hydroxychloroquine vs. active treatment

**Figure 3.**
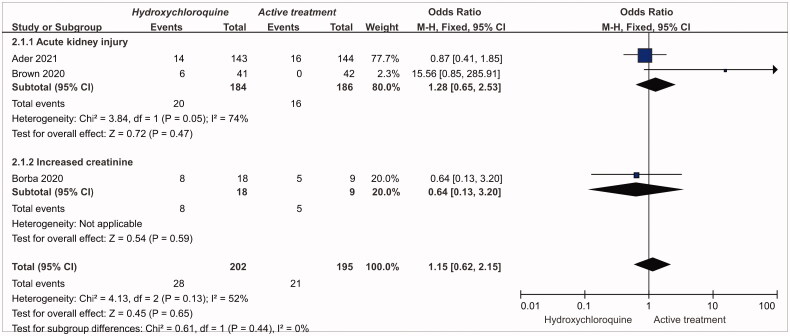
The risk of AKI and increased creatinine for hydroxychloroquine/chloroquine compared to active treatment based on the evidence of RCTs.

Two RCTs compared the risk of AKI, and one RCT compared the risk of increased creatinine between hydroxychloroquine/chloroquine and active treatment (Figure 3). Compared to active treatment, the meta-analysis showed that the OR of AKI with hydroxychloroquine/chloroquine was 1.28 (95%CI: 0.65, 2.53; *p* = .47, *n* = 2, low quality), with substantial heterogeneity (I^2^ = 74%), while the OR of increased creatinine was 0.64 (95%CI: 0.13, 3.2; *p* = .59, *n* = 1, low quality). However, heterogeneity was inestimable as only one study was available. Based on NRSIs, the OR was 1.43 (95%CI: 1.06, 1.94; *p* = .01, *n* = 8, low quality) for AKI with hydroxychloroquine/chloroquine, and 2.00 (95%CI: 0.45, 8.80; *p* = .36, *n* = 1, low quality) for increased creatinine, Figure S3 (Supplementary Appendix).

### Sensitivity analysis and publication bias

The results of the sensitivity analyses are presented in the Supplementary Appendix file (Figure S4 to S7), which showed that the effects remained stable in the random-effect model based on the evidence from RCTs. However, for evidence of NRSIs, the removal of the studies by Grimaldi [31] or Schneider [33] caused the pooled OR to no longer be significant (*p* > .05) comparing hydroxychloroquine/chloroquine and active treatment. However, this did not alter the direction of the effects.

Considering the limited number of studies on the two outcomes in the meta-analyses, we combined them to detect the potential publication bias, with our results suggesting absent bias (Egger’s *p* = .56 for meta-analysis based on RCTs for placebo; Egger’s *p* = .86 for meta-analysis based on NRSIs for active treatment).

## Discussion

We conducted a systematic review and meta-analysis to investigate the risk of AKI and increased creatinine levels in COVID-19 patients treated with hydroxychloroquine/chloroquine. To the best of our knowledge, this was the first systematic review to investigate the harmful effects of hydroxychloroquine/chloroquine in COVID-19 patients. Our results suggest that there is currently no strong evidence that hydroxychloroquine/chloroquine increases the risk of AKI in COVID-19 patients.

In our meta-analysis, the potential for increased risk of AKI was detected by evidence from cohort and case-control studies. However, the quality of the evidence was low, possibly due to the confounding factor that the majority of the NRSIs had a critical risk of bias from potential confounders. In addition, the unstable results in the post-hoc sensitivity analysis for the meta-analyses of NRSIs suggested some evidence of publication bias. We noticed that the sample size of Schneider’s study was small, while the effect was extremely large. Further, the study had a substantial impact on the pooled effects, which is clear evidence of publication bias. However, Egger’s test failed to confirm this.

Our study’s direct evidence suggests that the routine administration of hydroxychloroquine/chloroquine is not associated with nephrotoxicity in COVID-19 patients. Meanwhile, the indirect evidence suggests that other active treatments may not also result in nephrotoxicity in COVID-19 patients. This further indicates that the AKI cases reported in COVID-19 patients were more likely due to factors other than drug toxicity. Hirsch et al. [45] found that patients who required mechanical ventilation had an almost four times higher risk of developing AKI than non-ventilated patients. Chen et al. found that in hospitalized COVID-19 patients, a higher level of serum phosphorus was associated with an increased risk of AKI [46]. Further research on the molecular biology mechanism is helpful in exploring the potential relationship between COVID-19 infection and AKI.

The current study had some strengths. First, the current study used a systematic review approach to summarize all available studies to ensure more representative and credible results. Second, we also maintained a standard and strict process during the conduct of the systematic review and meta-analysis to guarantee the quality of the current study. Furthermore, the quality of the included RCTs and the homogenous effects ensure a moderate quality of evidence, making the findings conclusive. However, this study had some limitations. First, although we included both RCTs and NRSIs, the quality of the evidence from NRSIs was proved to be low, contributing little contribution to the conclusion. Furthermore, the analysis of NRSIs takes a large amount of time, which largely prolonged the conduct of the current systematic review. Second, there were only four or five RCTs for each outcome, and most of the RCTs were based on an open-label design, which may have led to some bias in the results. Therefore, we downgraded the evidence as moderate. Third, some RCTs reported limited information on the safety outcomes, excluding renal system adverse events, and therefore were not included in the current study. However, such RCTs may have collected the information, but the authors failed to report it, leading to a potential loss of evidence. These limitations should be addressed in updated meta-analyses.

In conclusion, based on currently available studies which were graded as low to moderate quality, there is insufficient evidence to conclude that hydroxychloroquine/chloroquine use is associated with increased risk of AKI or raised creatinine.

## Supplementary Material

Supplemental Material
