# Supplementary material for: Hydroxychloroquine/chloroquine and the risk of acute kidney injury in COVID-19 patients: a systematic review and meta-analysis
Source: Ren Fail. 2022 Mar 7;44(1):415–25. doi: 10.1080/0886022X.2022.2046609 (PMC8903764; doi:10.1080/0886022X.2022.2046609)

PubMed

((("Hydroxychloroquine"[MeSH Terms] OR "Hydroxychloroquine"[Title/Abstract] OR "Oxychloroquine"[Title/Abstract] OR "Plaquenil"[Title/Abstract] OR ("Chloroquine"[MeSH Terms] OR "Chloroquine"[Title/Abstract] OR "Chlorochin"[Title/Abstract] OR "Chingamin"[Title/Abstract] OR "Khingamin"[Title/Abstract] OR "Nivaquine"[Title/Abstract] OR "Aralen"[Title/Abstract] OR "Arechine"[Title/Abstract])) AND ("COVID-19"[MeSH Terms] OR "COVID-19"[Title/Abstract] OR "COVID19"[Title/Abstract] OR "COVID"[Title/Abstract] OR "2019-Cov"[Title/Abstract] OR "2019-nCov"[Title/Abstract] OR "nCov"[Title/Abstract] OR "severe acute respiratory syndrome coronavirus 2"[Title/Abstract] OR ("SARS-CoV-2"[MeSH Terms] OR "SARS-CoV-2"[Title/Abstract] OR "sars coronavirus 2"[Title/Abstract] OR "SARS-2"[Title/Abstract] OR "SARS2"[Title/Abstract] OR "severe acute respiratory syndrome coronavirus 2"[Title/Abstract] OR "2019-novel-corona"[Title/Abstract] OR "Novel-corona"[Title/Abstract] OR "novel coronavirus"[Title/Abstract] OR "New-corona"[Title/Abstract] OR "Coronavirus"[Title/Abstract] OR "coronavirus 2"[Title/Abstract] OR "corona virus"[Title/Abstract] OR "Betacoronavirus"[Title/Abstract] OR "SARS-2"[Title/Abstract] OR "Cov2"[Title/Abstract]))) NOT ("Review"[Publication Type] OR "systematic review"[Publication Type] OR "Meta-analysis"[Publication Type])) AND (humans[Filter])

Embase

No.	Query	Results	Date
#1	'chloroquine'/exp OR chloroquine	46430	10-Jul-21
#2	chloroquine:ti,ab	23483	10-Jul-21
#3	chlorochin:ti,ab	11	10-Jul-21
#4	chingamin:ti,ab	3	10-Jul-21
#5	khingamin:ti,ab	2	10-Jul-21
#6	nivaquine:ti,ab	126	10-Jul-21
#7	aralen:ti,ab	40	10-Jul-21
#8	arechine:ti,ab	4	10-Jul-21
#9	'hydroxychloroquine'/exp	33900	10-Jul-21
#10	hydroxychloroquine:ti,ab	11726	10-Jul-21
#11	oxychloroquine:ti,ab	8	10-Jul-21
#12	plaquenil:ti,ab	371	10-Jul-21
#13	#1 OR #2 OR #3 OR #4 OR #5 OR #6 OR #7 OR #8 OR #9 OR #10 OR #11 OR #12	75445	10-Jul-21
#14	'coronavirus disease 2019'/exp	128811	10-Jul-21
#15	covid19:ti,ab	1929	10-Jul-21
#16	covid:ti,ab	130746	10-Jul-21
#17	'2019 cov':ti,ab	16	10-Jul-21
#18	'2019 ncov':ti,ab	1314	10-Jul-21
#19	ncov:ti,ab	1570	10-Jul-21
#20	'sars cov 2'/exp	36004	10-Jul-21
#21	'sars cov 2':ti,ab	41592	10-Jul-21
#22	'sars coronavirus 2':ti,ab	250	10-Jul-21
#23	'sars 2':ti,ab	87	10-Jul-21
#24	'severe acute respiratory syndrome coronavirus 2':ti,ab	13431	10-Jul-21
#25	'2019 novel corona':ti,ab	26	10-Jul-21
#26	'novel corona':ti,ab	342	10-Jul-21
#27	novel AND coronavirus:ti,ab	13195	10-Jul-21
#28	'new corona':ti,ab	58	10-Jul-21
#29	coronavirus:ti,ab	61851	10-Jul-21
#30	'coronavirus 2':ti,ab	14405	10-Jul-21
#31	'corona virus':ti,ab	2160	10-Jul-21
#32	betacoronavirus:ti,ab	493	10-Jul-21
#33	'sars 2':ti,ab	87	10-Jul-21
#34	cov2:ti,ab	2182	10-Jul-21
#35	#14 OR #15 OR #16 OR #17 OR #18 OR #19 OR #20 OR #21 OR #22 OR #23 OR #24 OR #25 OR #26 OR #27 OR #28 OR #29 OR #30 OR #31 OR #32 OR #33 OR #34	171345	10-Jul-21
#36	#13 AND #35	8576	10-Jul-21
#37	review:it OR 'systematic review':it OR 'meta analysis':it OR letter:it OR 'conference abstract':it OR note:it OR 'conference	9653773	10-Jul-21

	review':it OR editorial:it OR chapter:it		
#38	#36 NOT #37	4533	10-Jul-21
#39	#38 AND 'human'/de	4296	10-Jul-21
#40	#38 AND 'human'/de AND ('clinical article'/de OR 'clinical study'/de OR 'clinical trial'/de OR 'clinical trial topic'/de OR 'cohort analysis'/de OR 'comparative study'/de OR 'controlled study'/de OR 'major clinical study'/de OR 'multicenter study'/de OR 'multicenter study topic'/de OR 'observational study'/de OR 'prospective study'/de OR 'randomized controlled trial'/de OR 'randomized controlled trial topic'/de OR 'retrospective study'/de)	3229	10-Jul-21

Figure S1. The flow plot.

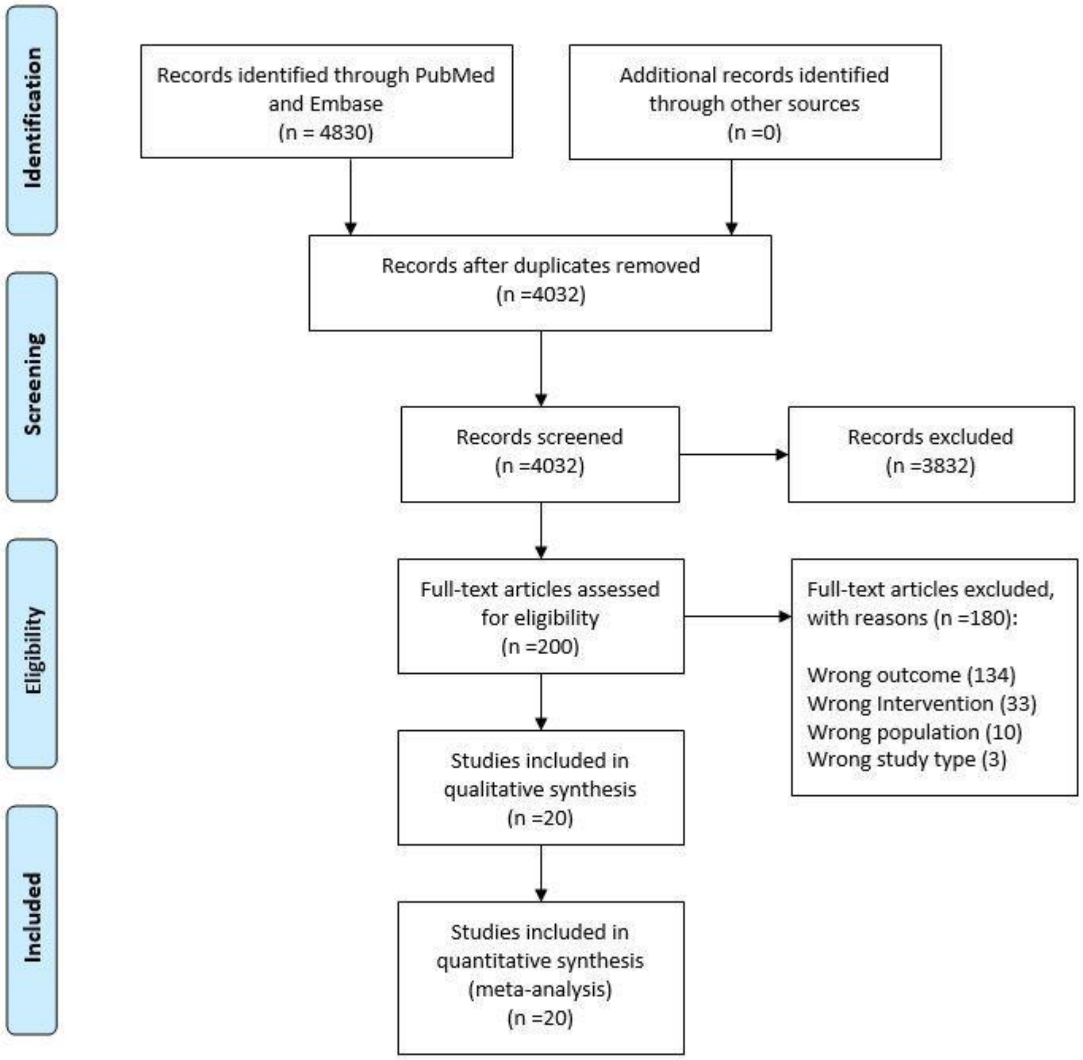

Figure S2. The risk of AKI and increased creatinine for hydroxychloroquine/chloroquine compared to placebo based on the evidence of NRSIs. (No studies for increased creatine)

	Hydroxychloroquine		Placebo			Odds ratio
Study or Subgroup	Events	Total	Events	Total	Weight	M-H, Fixed, 95% CI
**1.2.1 Acute kidney injury**						
Lagier 2020	0	101	0	137		Not estimable
Lecronier 2020	16	38	12	20	100.0%	0.48 [0.16 , 1.46]
**Subtotal (95% CI)**		**139**		**157**	**100.0%**	**0.48 [0.16 , 1.46]**
Total events:	16		12			
Heterogeneity: Not applicable						
Test for overall effect: Z = 1.29 (P = 0.20)						
**Total (95% CI)**		**139**		**157**	**100.0%**	**0.48 [0.16 , 1.46]**
Total events:	16		12			
Heterogeneity: Not applicable						
Test for overall effect: Z = 1.29 (P = 0.20)						
Test for subgroup differences: Not applicable						

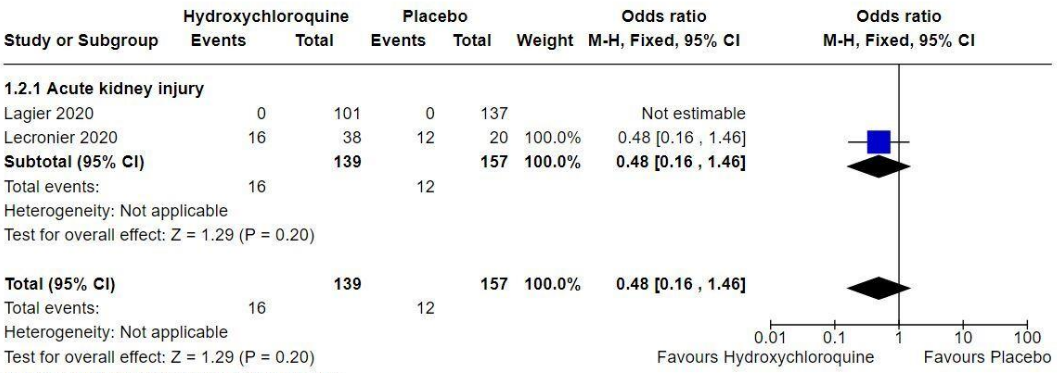

Figure S3. The risk of AKI and increased creatinine for hydroxychloroquine/chloroquine compared to active treatment based on the evidence of NRSIs.

	Hydroxychloroquine		Active treatment			Odds ratio
Study or Subgroup	Events	Total	Events	Total	Weight	M-H, Fixed, 95% CI
**2.2.1 Acute kidney injury**						
Basalely 2021	3	8	14	88	2.0%	3.17 [0.68 , 14.81]
Falcão 2021	8	101	1	48	1.7%	4.04 [0.49 , 33.29]
Ghosn 2021	18	50	27	60	21.7%	0.69 [0.32 , 1.48]
Grimaldi 2020	124	183	137	231	53.9%	1.44 [0.96 , 2.16]
Lagier 2020	4	3337	0	137	1.3%	0.37 [0.02 , 6.93]
Lecronier 2020	16	38	12	22	12.2%	0.61 [0.21 , 1.75]
Pham 2021	9	14	9	28	3.0%	3.80 [0.98 , 14.67]
Schneider 2020	11	14	2	14	0.6%	22.00 [3.08 , 157.34]
**Subtotal (95% CI)**		**3745**		**628**	**96.4%**	**1.43 [1.06 , 1.94]**
Total events:	193		202			
Heterogeneity: Chi² = 18.22, df = 7 (P = 0.01); I² = 62%						
Test for overall effect: Z = 2.32 (P = 0.02)						
**2.2.2 Increased Creatinine**						
Kim 2021	6	34	3	31	3.6%	2.00 [0.45 , 8.80]
**Subtotal (95% CI)**		**34**		**31**	**3.6%**	**2.00 [0.45 , 8.80]**
Total events:	6		3			
Heterogeneity: Not applicable						
Test for overall effect: Z = 0.92 (P = 0.36)						
**Total (95% CI)**		**3779**		**659**	**100.0%**	**1.45 [1.08 , 1.96]**
Total events:	199		205			
Heterogeneity: Chi² = 18.44, df = 8 (P = 0.02); I² = 57%						
Test for overall effect: Z = 2.46 (P = 0.01)						
Test for subgroup differences: Chi² = 0.19, df = 1 (P = 0.67), I² = 0%						

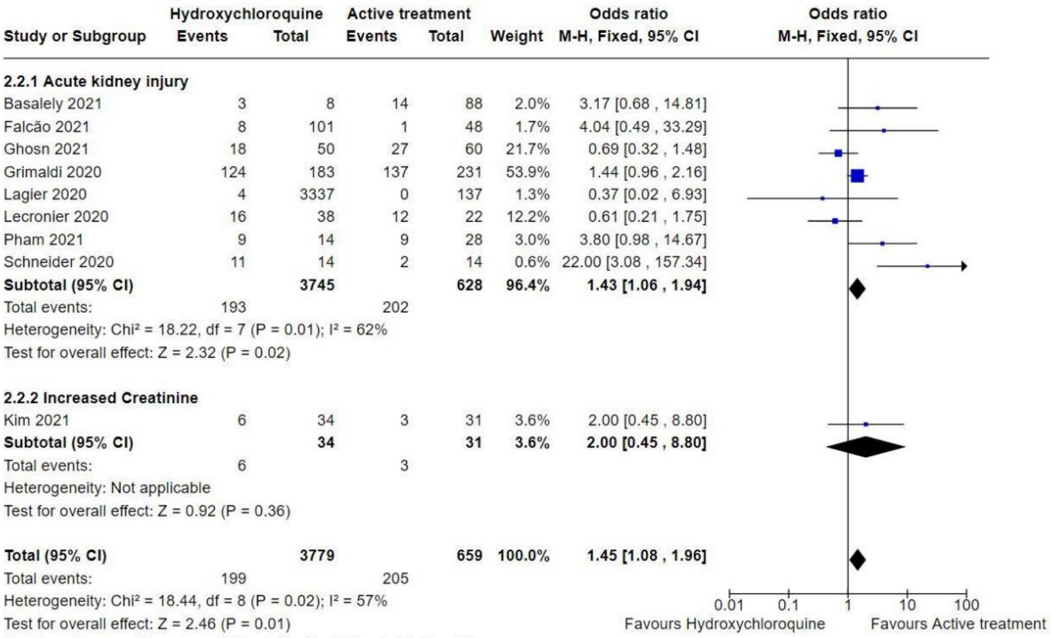

Figure S4. Sensitivity analysis by random-effect model (Hydroxychloroquine vs. active treatment, RCTs).

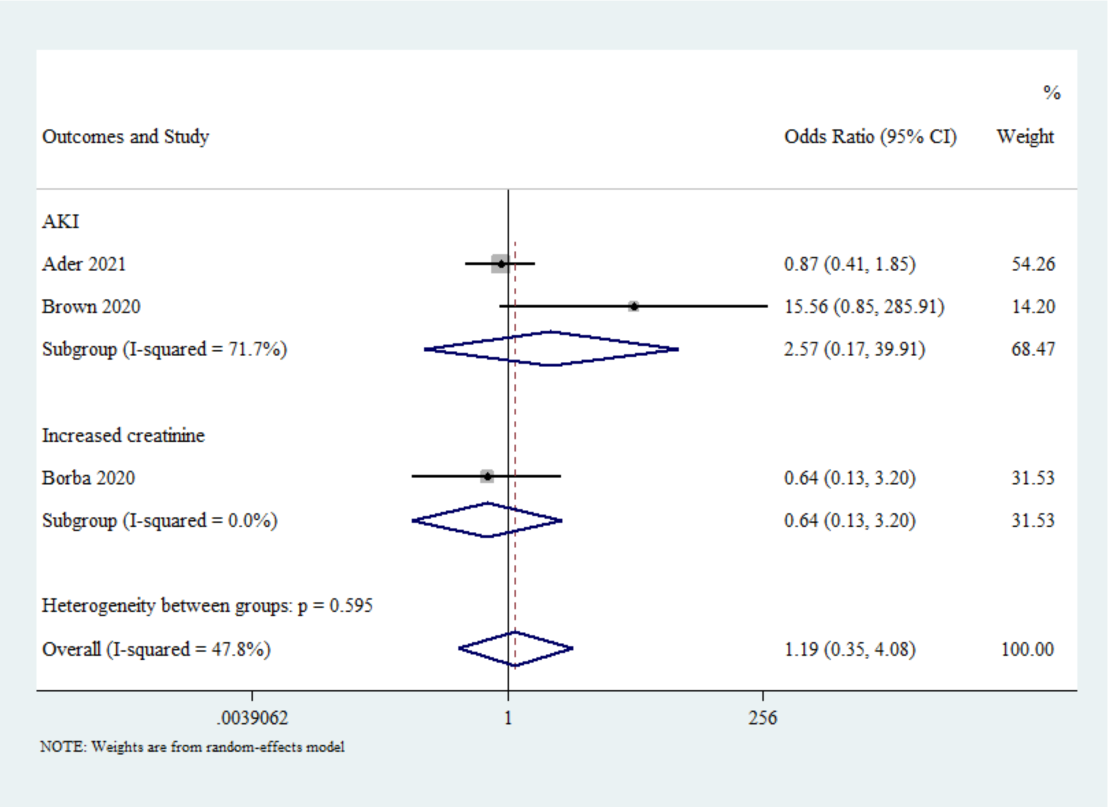

Figure S5. Sensitivity analysis by random-effect model (Hydroxychloroquine vs. active treatment, NRSIs).

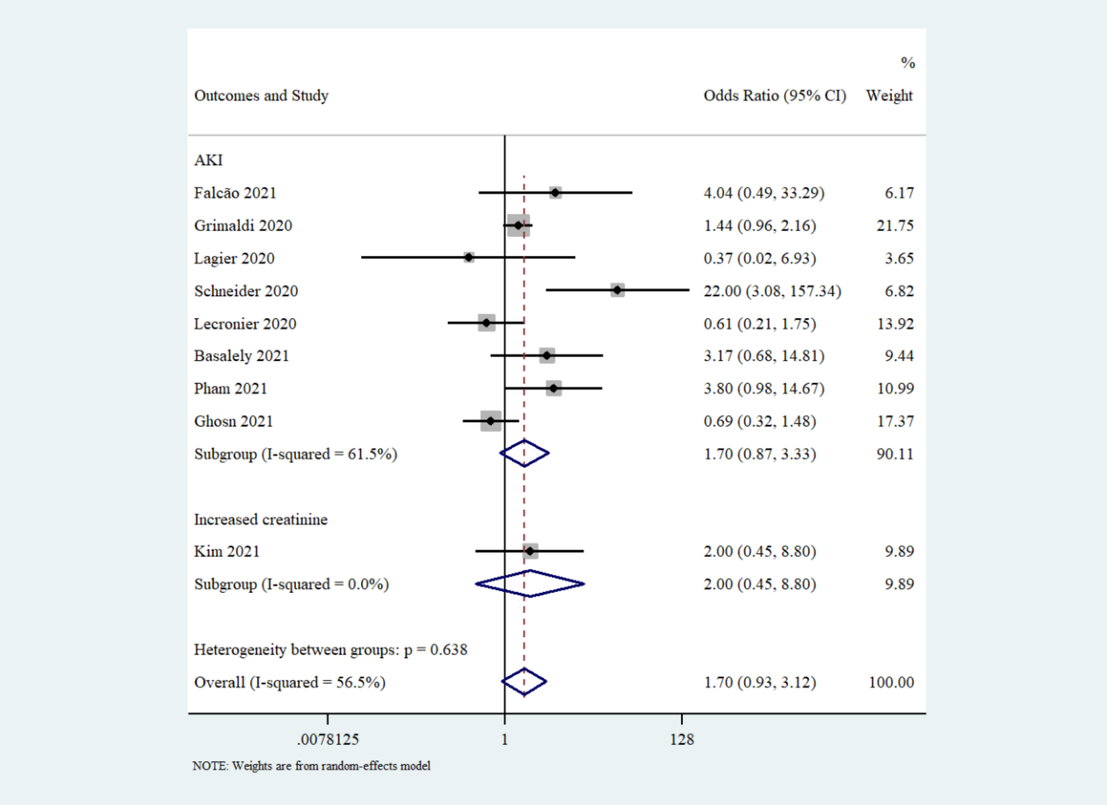

Figure S6. Sensitivity analysis by MH RD.

Study or Subgroup	Hydroxychloroquine Events	Hydroxychloroquine Total	Placebo Events	Placebo Total	Weight	Risk difference M-H, Fixed, 95% CI
Lagier 2020	0	101	0	137	81.6%	0.00 [-0.02 , 0.02]
Lecronier 2020	16	38	12	20	18.4%	-0.18 [-0.44 , 0.09]
**Total (95% CI)**		**139**		**157**	**100.0%**	**-0.03 [-0.08 , 0.02]**
Total events:	16		12			

Heterogeneity: Chi² = 15.80, df = 1 (P < 0.0001); I² = 94%
Test for overall effect: Z = 1.27 (P = 0.20)
Test for subgroup differences: Not applicable

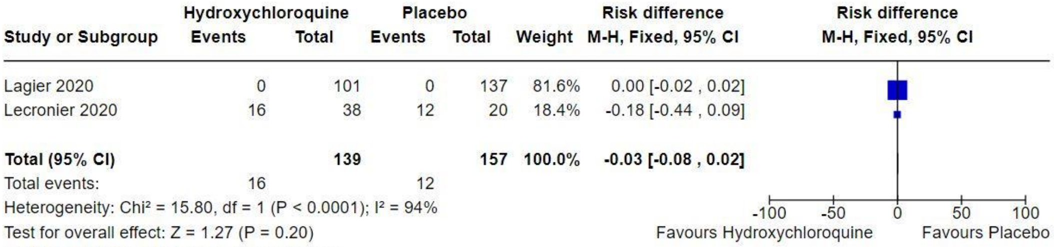

Figure S7. Sensitivity analysis by omitting one study at a time showed unstable results (Hydroxychloroquine vs. active treatment, NRSIs).

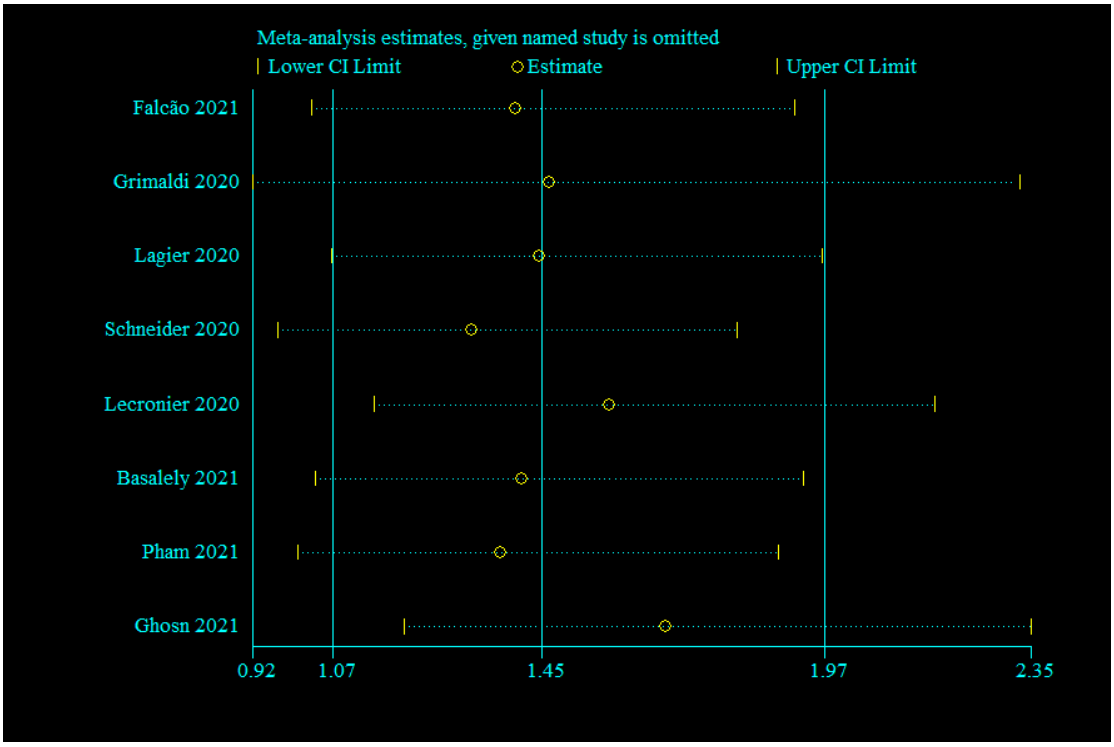

Supplement: Supplemental Material [file IRNF_A_2046609_SM0669.pdf]
